# The Effect of OPA1 on Mitochondrial Ca^2+^ Signaling

**DOI:** 10.1371/journal.pone.0025199

**Published:** 2011-09-29

**Authors:** László Fülöp, Gergö Szanda, Balázs Enyedi, Péter Várnai, András Spät

**Affiliations:** 1 Department of Physiology, Faculty of Medicine, Semmelweis University, Hungarian Academy of Sciences, Budapest, Hungary; 2 Laboratory of Neurobiochemistry and Molecular Physiology, Hungarian Academy of Sciences, Budapest, Hungary; Karolinska Institutet, Sweden

## Abstract

The dynamin-related GTPase protein OPA1, localized in the intermembrane space and tethered to the inner membrane of mitochondria, participates in the fusion of these organelles. Its mutation is the most prevalent cause of Autosomal Dominant Optic Atrophy. OPA1 controls the diameter of the junctions between the boundary part of the inner membrane and the membrane of cristae and reduces the diffusibility of cytochrome *c* through these junctions. We postulated that if significant Ca^2+^ uptake into the matrix occurs from the lumen of the cristae, reduced expression of *OPA1* would increase the access of Ca^2+^ to the transporters in the crista membrane and thus would enhance Ca^2+^ uptake. In intact H295R adrenocortical and HeLa cells cytosolic Ca^2+^ signals evoked with K^+^ and histamine, respectively, were transferred into the mitochondria. The rate and amplitude of mitochondrial [Ca^2+^] rise (followed with confocal laser scanning microscopy and FRET measurements with fluorescent wide-field microscopy) were increased after knockdown of *OPA1*, as compared with cells transfected with control RNA or *mitofusin1* siRNA. Ca^2+^ uptake was enhanced despite reduced mitochondrial membrane potential. In permeabilized cells the rate of Ca^2+^ uptake by depolarized mitochondria was also increased in *OPA1*-silenced cells. The participation of Na^+^/Ca^2+^ and Ca^2+^/H^+^ antiporters in this transport process is indicated by pharmacological data. Altogether, our observations reveal the significance of OPA1 in the control of mitochondrial Ca^2+^ metabolism.

## Introduction

Recent observations obtained in imaging and electron tomographic studies revealed a dynamically changing structure [1], [2] and led to a revised concept of the structure and function of mitochondria. The changes in the number and size of mitochondria involve alterations in the inner mitochondrial membrane (IMM). The invaginations of the IMM, termed cristae, display various conformations under changing energetic conditions. The cristae are connected to the inner boundary membrane (i.e. the part of IMM between two neighboring cristae) by narrow tubular junctions which have a diameter of 15–40 nm [3]–[6]. These junctions may impede free diffusion and thus may induce the formation of a gradient of ions, molecules and macromolecules between the intermembrane space (IMS) and the lumen of the cristae [5], [7].

The fusion of mitochondria is regulated by the transmembrane GTPase proteins mitofusin (Mfn) 1 and 2 and OPA1 [1]. Mutation of the *OPA1* gene is the most prevalent cause of the type 1 Autosomal Dominant Optic Atrophy [8], [9]. Although the fundamental pathology is the degeneration of retinal ganglion cells with subsequent atrophy of the optic nerve [1], [10], [11], the protein is expressed in all examined human tissues, explaining the accidental association of blindness with external ophthalmoplegia and various neuromuscular lesions [12]–[14]. Reduced expression of OPA1 was also reported in ischaemic heart failure [15] showing that insufficient expression of the protein may have far-reaching consequences.

OPA1 (and Mgm1, its ortholog in the yeast) is a dynamin-related GTPase protein. Due to alternative splicing, its gene is transcribed into 8 mRNA isoforms [16]. The protein is tethered to the IMM [17]–[19] and localized in the IMS [18], [20]. In Western blot analysis 5 separate bands (designated *a* to *e*) of molecular weight, ranging from 94 to 86 kDa, can be found. Two long isoforms are anchored to the IMM and three soluble short forms are located in the IMS. These latter bands are the proteolytic products of the long forms [21], [22]. Heteromultimeric complex formation of Opa1 was suggested on the basis of the relatively constant stoichiometry of the long and short isoforms [23]. On their own long and short isoforms have little impact on the fusion of mitochondria, but when coexpressed they functionally complement one another [24].

OPA1 regulates the diameter of the crista junction. The tightness of junctions correlates with the oligomerization of the membrane-bound and the soluble forms in the IMS [25]. Knockdown of either the *Mfn1* [26]–[28] or the *OPA1* gene brings about the fragmentation of the mitochondria [19], [29], [30], moreover, knocking down of *OPA1* (or *Mgm1*) gene also evokes drastic desorganisation of the cristae [29], [31]–[34]. An essential component of the structural change is the dilation of the junctions [4] (but see [6]). It has been proposed several years ago that the size of the junction may modify the diffusion of molecules like adenine nucleotides [3].

The role of the junction in the control of mitochondrial metabolism has been suggested by numerous observations. The majority of cytochrome *c* reductase, F_1_F_0_ ATPase [35] and of cytochrome *c* oxidase [36], as well as the uncoupling protein 1 (in brown adipocytes) [37] are found within the crista membrane. Only 10–15% of cytochrome *c* is found free in the IMS, while the major fraction can be found in the cristae [4], [38]. The proapoptotic agent truncated Bid (t-Bid) known to evoke disassembly of OPA1 oligomers [6], [25] brought about a drastic increase in the junction diameter [4]. This increase was associated with enhanced cytochrome c release from digitonin-permeabilized mitochondria [4], [6]. The effect of t-Bid could be potentiated with silencing of *OPA1* gene [33] and overcome with overexpression of OPA1 [25], indicating that the clearance of the junction is controlled by OPA1. These observations strongly suggest that the state of OPA1 may modify substrate-dependent enzymatic and transport processes occuring in the crista membrane.

Calcium mobilising agonists generate cytosolic Ca^2+^ signal that is rapidly transferred into the mitochondrial matrix. This sequestration of Ca^2+^ shapes the cytosolic Ca^2+^ signal and thereby modifies all the Ca^2+^ transport processes. The mitochondrial Ca^2+^ signal enhances the reduction of mitochondrial pyridine nucleotides and thus potentiates ATP formation (reviewed e.g. in [39]) and hormone secretion [40]. Out of various mechanisms responsible for Ca^2+^ transport from the IMS into the mitochondrial matrix (reviewed in [41]–[43]) the most important one is the ruthenium red-sensitive mitochondrial Ca^2+^ uniporter (MCU), the driving force of the transport is the mitochondrial membrane potential (∼ 180 mV, inside negative) (reviewed in [41]). Patch-clamp studies revealed that the uniporter is an inwardly rectifying cation channel [44], [45] and a 40 kDa membrane protein has recently been shown to exhibit the conductive function [46], [47]. (A recently characterized protein, MiCu1 seems to be its Ca^2+^-sensing subunit [48]). The electrogenic mitochondrial Na^+^/Ca^2+^ antiporter was also reported to transport Ca^2+^ into the mitochondria [49], [50]. Similarly, the electrogenic Ca^2+^/H^+^ antiporter, identified as Letm1 [51] may also be responsible for Ca^2+^ uptake by depolarized mitochondria. In spite of the progress in the elucidation of the structure and function of the transporters there are no data available on their location within the IMM. The only available exception is the demonstration of mitochondrial Na^+^/Ca^2+^ exchanger (NCLX) in the crista membrane with immunoelectron microscopy [52]. Nevertheless, schematic drawings in several recently published reviews locate the transporters in the boundary membrane and no Ca^2+^ transport is indicated within the cristae [42], [43], [53]–[56]. Here we report that the expression of OPA1 modifies mitochondrial Ca^2+^ uptake, suggesting the significance of the crista membrane in Ca^2+^ uptake. Our results imply that OPA1 may be a target of factors controlling mitochondrial Ca^2+^ metabolism.

## Results

### Mitochondrial morphology in *OPA1* siRNA-transfected cells

**Figure 1:**
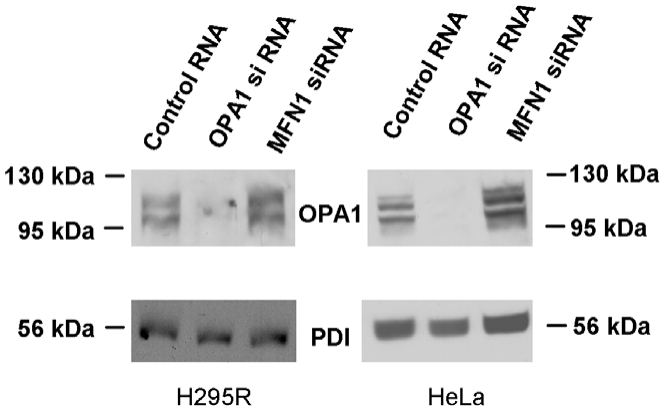
Effect of *OPA1* and *Mfn1* siRNA on the expression of OPA1. The cells were transfected with *OPA1* siRNA, *Mfn1* siRNA or a non-silencing RNA on the day following plating (day 2). The samples were lysed on day 5, run on SDS-PAGE, transferred onto nitrocellulose membrane, incubated with anti-OPA1 mouse monoclonal antibody then with anti-mouse immunoglobulin-horseradish-peroxidase conjugate. Protein disulphide isomerase was used as loading control.

In order to evaluate the effect of OPA1 on mitochondrial Ca^2+^ uptake we depressed its expression with siRNA. Since silencing of *OPA1* has been known to evoke fragmentation of the mitochondria (see Introduction) and the ensuing increase in surface/volume ratio might accelerate the increase in [Ca^2+^]_m_, a group of cells was treated with *Mfn1* siRNA with the intention of evoking similar mitochondrial fragmentation. Western blot analysis confirmed the specificity of the *OPA1* siRNA on the expression of OPA1 (Figure 1). (The moderate reduction of protein expression in H295R cells may be accounted for by the poor transfectability of this cell line.) To test whether *Mfn1*-silenced cells are more appropriate controls for *OPA1*-silenced cells than those transfected with control (non-silencing) RNA, the morphology of mitochondria was compared in the three groups. As shown in Figures S1 and S2 for H295R and HeLa cells, the filamentous mitochondrial pattern in cells exposed to control RNA changed into fragmented, round-shaped mitochondia in *OPA1* and *Mfn1*-silenced cells. In H295R cells the median value of the length of single mitochondria diminished from 2.30 µm in control RNA-treated cells to 1.08 and 0.98 µm in cells exposed to *Mfn1* and *OPA1* siRNA, respectively (Figure S3). In HeLa cells the median lenght changed from 2.73 to 0.40 and 0.24 µm, respectively (Figure S3). Another conventional indicator of fragmentation, the circularity (for a circle its value is 1) increased from a median of 0.199 to 0.447 and 0.452 in H295R cells and from 0.217 to 0.743 and 0.803 in HeLa cells (Figure S3). The comparable values obtained in *OPA1* and *Mfn1* siRNA-treated cells show that the latter one is an appropriate control for studying the effect of OPA1 on Ca^2+^ metabolism.

### Effect of OPA1 silencing on mitochondrial Ca^2+^ uptake in intact cells

For monitoring cytosolic Ca^2+^ responses the fluorescent dye Fura-2 or Fura-FF was used. For following changes in mitochondrial [Ca^2+^] ([Ca^2+^]_m_) in intact cells the FRET-based, mitochondrially targeted, Cameleon-derived fluorescent protein 4mt-D1-cpV [57] or 4mt-D2-cpV [58], also targeted into the mitochondria, was applied. Their K_d_ for Ca^2+^ was about 10 µM and 85 nM, respectively. (Using null-point titration we measured pH∼8.0 in the mitochondria of resting HeLa and H295R cells, therefore the K_d_ values were determined at pH 8.0. ). The FRET ratio of the fluorescent proteins was insensitive to pH in the 7.6–8.2 range (data not shown). Fura-2 and 4mt-D2-cpV proved to be the appropriate sensors in H295R cells whereas Fura-FF and 4mt-D1-pcV were applied in HeLa cells.

**Figure 2:**
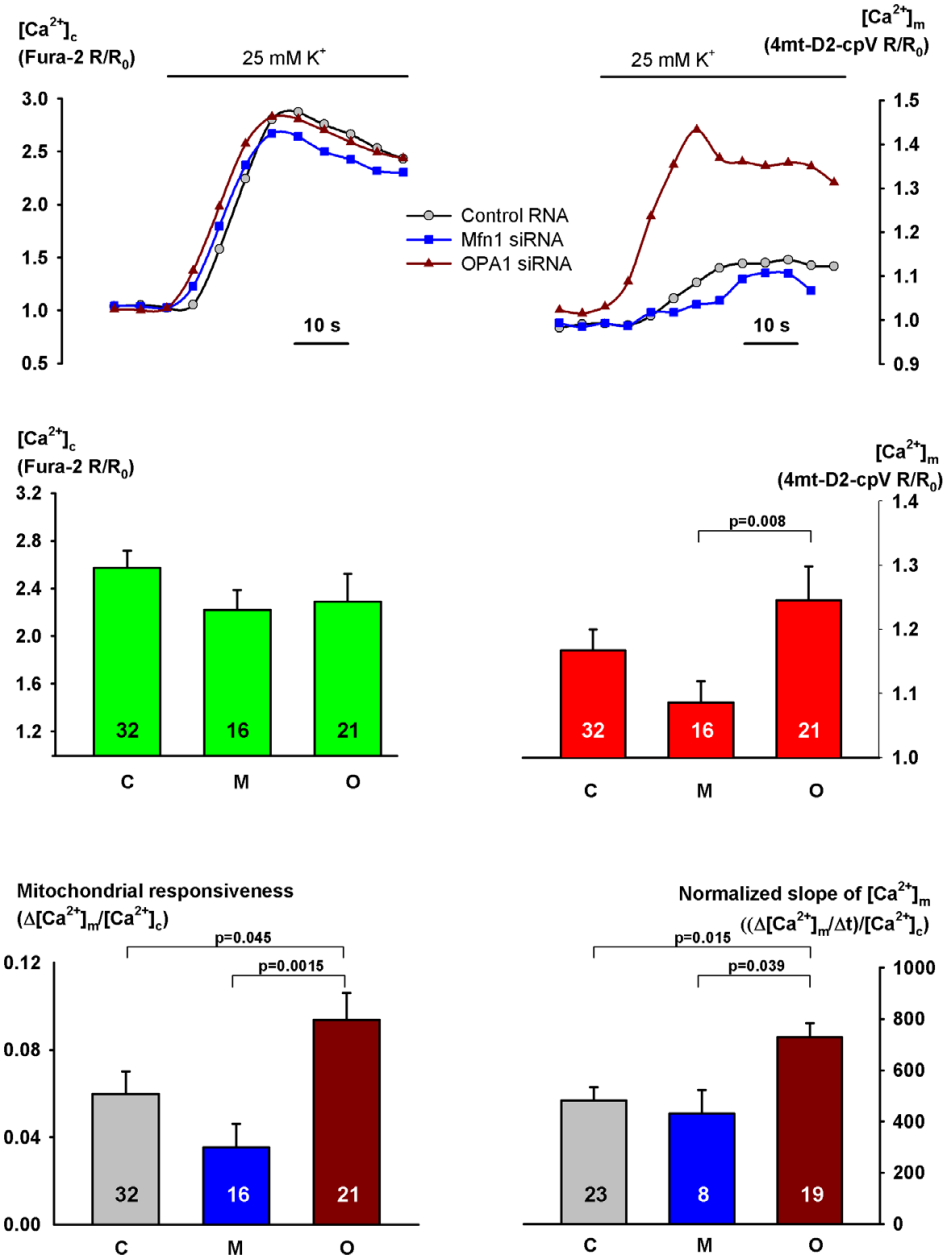
Effect of *OPA1* or *Mfn1* silencing on Ca^2+^ signaling in intact H295R cells. The cells were transfected with control RNA or siRNA and 4mt-D2-cpV on the day following plating (day 2)and once again with control RNA or siRNA on day 3. On day 5, after preloading with Fura-2 AM, the cells were stimulated with 25 mM K^+^. Changes in cytosolic [Ca^2+^] ([Ca^2+^]_c_) were monitored by measuring Fura-2 excitation ratio while [Ca^2+^]_m_ was indicated by the FRET ratio of 4mt-D2-cpV. Both ratios are normalized to those obtained in the control period. Representative cytosolic Ca^2+^ signals (**A**) and mitochondrial Ca^2+^ uptake curves (**B**) are shown for cells transfected with control RNA, *Mfn1* or *OPA1* siRNA. **C**: peak [Ca^2+^]_c_, **D**: peak [Ca^2+^]_m_ **E**: [Ca^2+^]_m_ response normalized to peak [Ca^2+^]_c_ (Δ[Ca^2+^]_m_/[Ca^2+^]_c_) indicating mitochondrial responsiveness; **F**: the slope of [Ca^2+^]_m_ rise related to peak [Ca^2+^]_c_ (cells not displaying a mitochondrial Ca^2+^ response were omitted from this statistics). Data are shown for control (C), *Mfn1* siRNA (M) or *OPA1* siRNA-transfected (O) groups. Results represent mean + SEM, the number of observations is shown within the columns.

In intact H295R cells we examined the transfer of cytosolic Ca^2+^ signal, elicited with 25 mM K^+^, into the mitochondrial matrix. Whereas the cytosolic signals were almost identical in the *Mfn1* and *OPA1* siRNA-treated cells, the FRET ratio of 4mt-D2-pcV, reflecting [Ca^2+^]_m_, showed a significant increase in the latter group (p =  0.008, Figure 2). When the mitochondrial Ca^2+^ response (Δ[Ca^2+^]_m_) was related to peak [Ca^2+^]_c_, the normalized mitochondrial response was again significantly higher in the *OPA1*-silenced cells (p = 0.0015). The rate of mitochondrial Ca^2+^ uptake (indicated by the slope of the FRET ratio increase) showed a considerable variance, however, when it was analyzed in function of [Ca^2+^]_c_, it was found to be significantly higher in the *OPA1*-silenced than in the *Mfn1*-silenced cells (p = 0.039) (Figure 2). (No detectable mitochondrial Ca^2+^ signal followed the cytosolic signal in a few cells, these values have been omitted from this statistics.)

**Figure 3:**
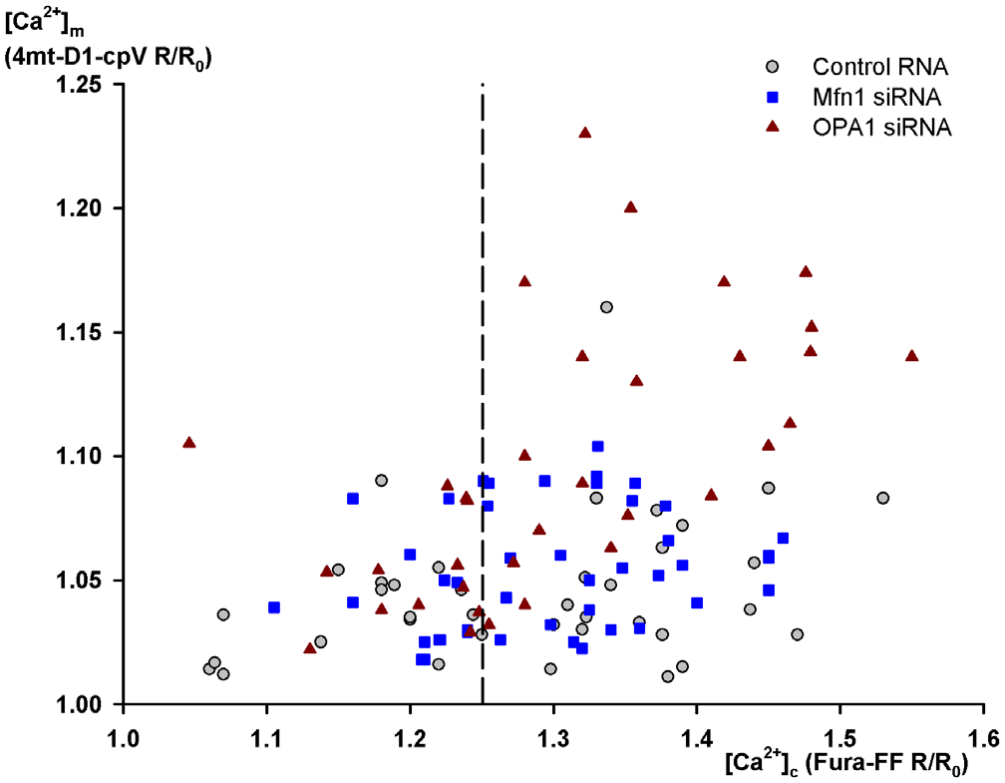
Effect of OPA1 or Mfn1 knockdown on Ca^2+^ signaling in intact HeLa cells. The cells were transfected with control RNA or siRNA on the day following plating (day 2) and with 4mt-D1-cpV on day 3. On day 5, after preloading with Fura-FF AM, the cells were stimulated with 1, 5 or 50 µM histamine. Changes in [Ca^2+^]_c_ were monitored by measuring Fura-FF excitation ratio while [Ca^2+^]_m_ was indicated by the FRET ratio of 4mt-D1-pcV. Both ratios are normalized to the values obtained in the control period. [Ca^2+^]_m_ for the pooled data, as indicated by the FRET signal of 4mt-D2-cpV, is shown in function of [Ca^2+^]_c_. Figure 4 contains statistics for the cells displaying normalized Fura-FF ratios greater than 1.25 (i.e. for the data shown right to the broken vertical line).

**Figure 4:**
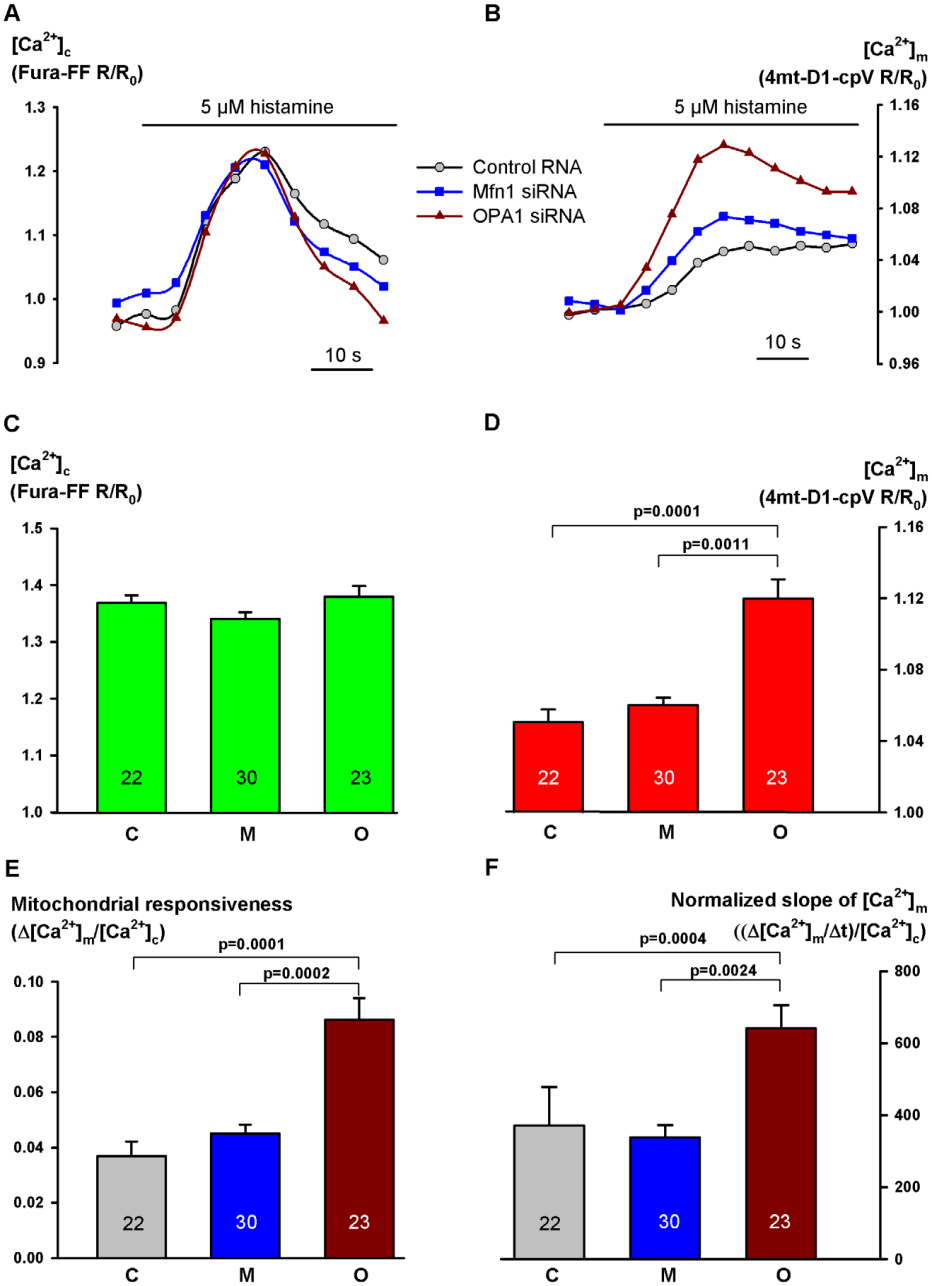
Mitochondrial Ca^2+^ signaling in intact, *OPA1* or *Mfn1* siRNA-treated HeLa cells displaying large [Ca^2+^]_c_ peak. (displaying normalized Fura-FF ratios greater than 1.25), shown in Figure 3 right to the broken vertical line. Representative curves are shown for [Ca^2+^]_c_ measured with Fura-FF (**A**) and for [Ca^2+^]_m_ measured with 4mt-D1-cpV (**B**) after stimulation with 5 µM histamine in cells transfected with control RNA, *Mfn1* siRNA or *OPA1* siRNA. Statistics for peak [Ca^2+^]_c_ (**C**), peak [Ca^2+^]_m_ (**D**), Δ[Ca^2+^]_m_/[Ca^2+^]_c_ indicating mitochondrial responsiveness (**E**) and the slope of [Ca^2+^]_m_ rise related to peak [Ca^2+^]_c_ (**F**) are shown for control (C), *Mfn1* siRNA (M) or *OPA1* siRNA-transfected (O) groups. Results represent mean + SEM, the number of observations is shown within the columns.

Intact HeLa cells were stimulated with histamine (1, 5 or 50 µM). While transfection with various RNAs did not influence the cytosolic Ca^2+^ response, *OPA1* siRNA enhanced the increase in [Ca^2+^]_m_ as compared with either the control RNA or the *Mfn1* siRNA-treated group. However, surprisingly, this effect was detectable only in cases of higher cytosolic response (Figure 3). When statistics was confined to the populations showing an R/R_o_ value (of Fura-FF)>1.25, the [Ca^2+^]_m_ peak, Δ[Ca^2+^]_m_ normalized to [Ca^2+^]_c_ peak and the slope of [Ca^2+^]_m_ increase normalized to peak [Ca^2+^]_c_ were all significantly greater in *OPA1*-silenced than in *Mfn1*-silenced cells (p =  0.0001, 0.0002, and 0.0024, resp.) (Figure 4).

### Mitochondrial membrane potential after gene silencing

Enhanced mitochondrial Ca^2+^ uptake can be attributed to increased mitochondrial membrane potential (Ψ_m_), increased activity of the Ca^2+^ transporting system as well as enhanced access of Ca^2+^ to the transporter. Assessment of Ψ_m_ with tetramethyl rhodamine methylester (TMRM) in H295R cells revealed that mitochondria were depolarized in *OPA1* siRNA-transfected cells as opposed to the effect of *Mfn1* siRNA (p = 5×10^−7^, Figure S4), a change attenuating rather than enhancing Ca^2+^ uptake. *OPA1* siRNA reduced Ψ_m_ examined with tetramethyl rhodamine ethylester (TMRE) also in HeLa cells (p = 0.0001). The reduction of Ψ_m_ has been confirmed with JC-1 (5,5′,6,6′-tetrachloro-1,1′,3,3′- tetraethyl-benzimidazolyl-carbocyanine iodide) (p = 0.0001, Figure S4).

### Effect of OPA1 silencing on mitochondrial Ca^2+^ uptake in permeabilized cells

**Figure 5:**
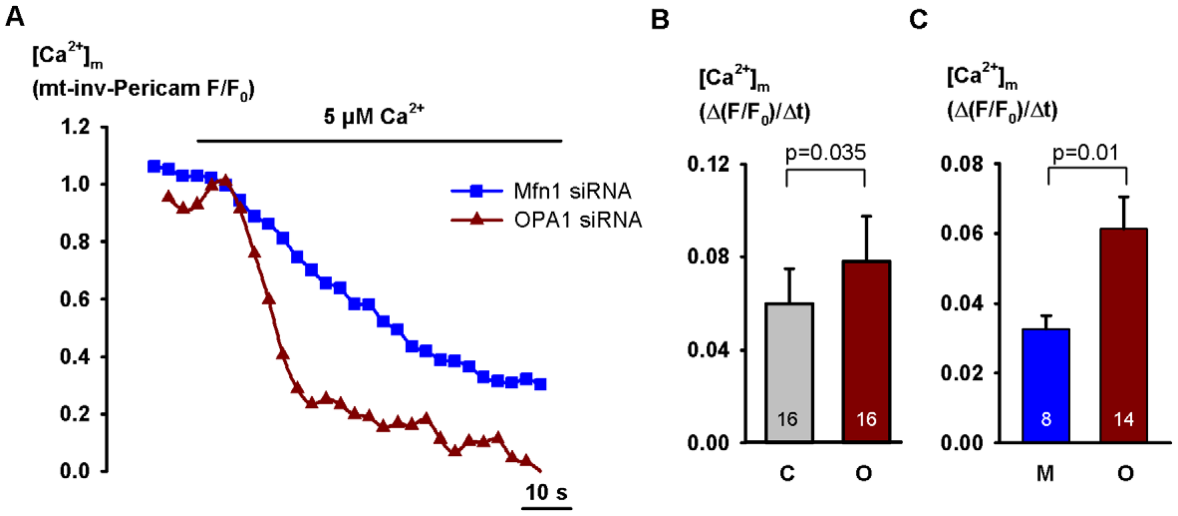
Mitochondrial Ca^2+^ uptake in permeabilized H295R cells transfected with *OPA1* or *Mfn1* siRNA. The cells were transfected with control RNA or siRNA on the day following plating (day 2) and with inverse Pericam targetted into the mitochondria (mt-inv-Pericam) on day 2 or 3. On day 5 the cells were permeabilized, superfused with a cytosol-like medium, Ψ_m_ was dissipated with 10 µM rotenon, 8 µg/ml oligomycin, 10 µM FCCP and 50 ng/ml valinomycin for 2 minutes. Then, in the presence of the drugs, [Ca^2+^] was raised from 0 to 5 µM. [Ca^2+^]_m_ was monitored by means of confocal microscopy, applying mt-inv-Pericam, the fluorescence of which exhibits inverse correlation with [Ca^2+^]. Fluorescence measured at saturating [Ca^2+^] (F_min_) was subtracted from each fluorescence value. The data were normalized for the control period. Representative mitochondrial Ca^2+^ uptake curves are shown for cells transfected with *Mfn1* or *OPA1* siRNA (note that decreasing F/F_0_ values indicate increasing [Ca^2+^]_m_!) (**A**); effect of *OPA1* siRNA as compared with that of control RNA (**B**) or *Mfn1* siRNA (**C**) on the slope of initial decrease of normalized mt-inv-Pericam fluorescence (indicating the slope of initial increase in [Ca^2+^]_m_). Results represent mean + SEM, the number of observations is shown within the columns.

**Figure 6:**
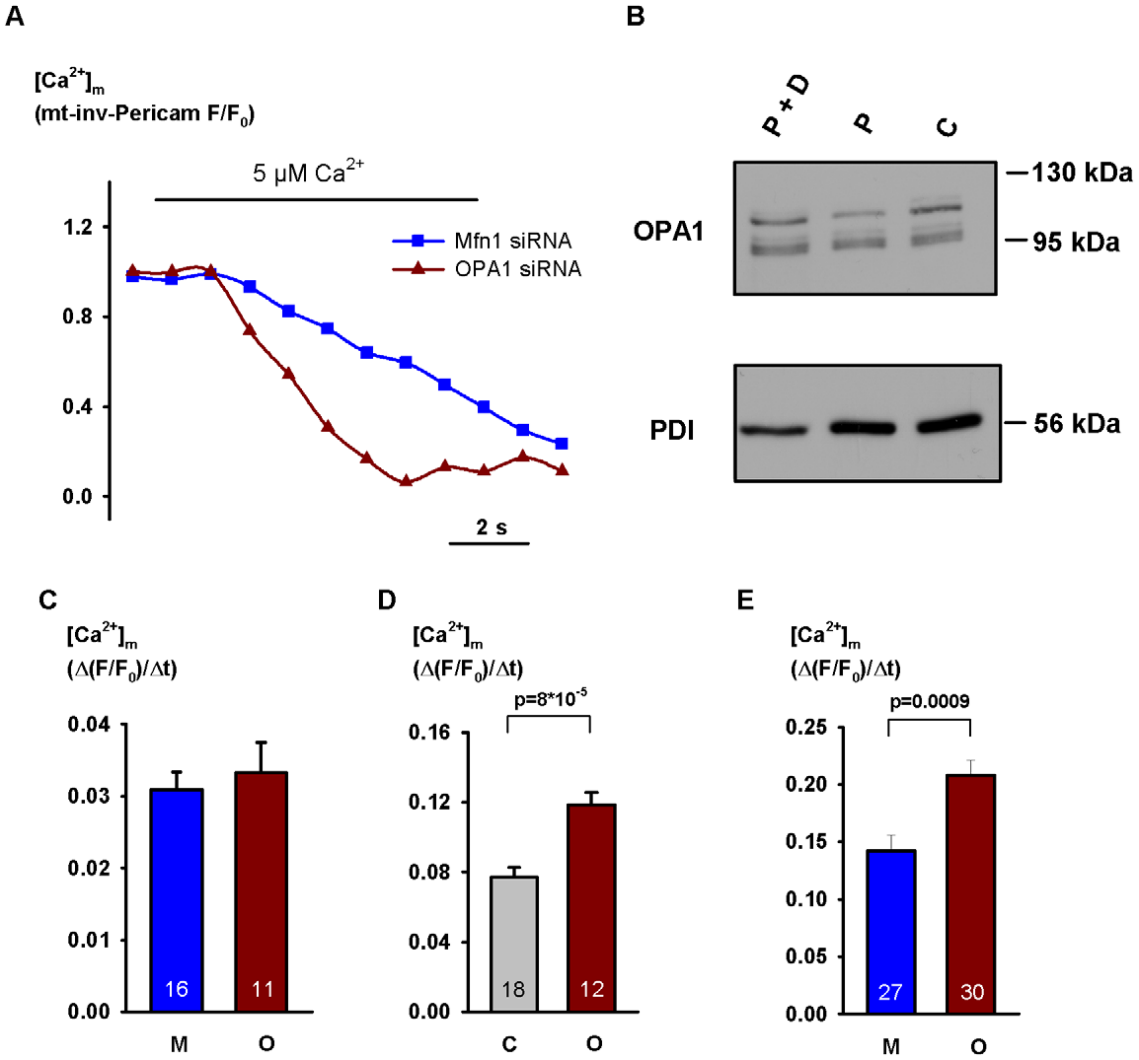
Effect of *OPA1* or *Mfn1* silencing on mitochondrial Ca^2+^ uptake in permeabilized HeLa cells. For the transfection protocol and measurement of fluorescence see legend of Figure 5. On day 5, after the dissipation of Ψ_m_ (see legend of Figure 5), [Ca^2+^] of the superfusion medium was raised from 0 to 2 or 5 µM. **A**: mitochondrial response to 5 µM Ca^2+^. Representative curves are shown for cells transfected with *Mfn* or *OPA1* siRNA (note that decreasing F/F_0_ values indicating increasing [Ca^2+^]_m_!). **B**: Western blot shows that 2-min exposure to the depolarizing medium did not change the pattern of immunoreactive OPA1. P: permeabilisation, D: dissipation of Ψ_m_, **C**: control. The slope of initial increase in [Ca^2+^]_m_ in permeabilized cells superfused with 2 µM (C) or 5 µM Ca^2+^ (**D** and **E**) is shown in cells transfected with control RNA (**D**), *Mfn1* siRNA (**C** and **E**) and *OPA1* siRNA (**C, D, E**). C: control RNA, M: *Mfn1* siRNA, O: *OPA1* siRNA. Results represent mean + SEM, the number of observations is shown within the columns.

In order to exclude extramitochondrial sites of OPA1 action and mostly to ensure identical driving force in each experimental group we next examined the effect of gene silencing in digitonin-permeabilized cells. The cells had been transfected with mitochondrially targeted inverse Pericam (mt-inv-Pericam). Its high Ca^2+^ affinity (K_d_ = 0.2 µM at pH 7.4 [59] and ∼80 nM at pH 8.0; not shown) rendered it an appropriate sensor for measuring *initial* Ca^2+^ uptake rate but no data could be obtained for the amplitude of the Ca^2+^ response. Following the permeabilization the cells were depolarized with a Ca^2+^-free cytosol-like medium lacking mitochondrial substrates but completed with 10 µM rotenon, 8 µg/ml oligomycin, 10 µM FCCP and 50 ng/ml valinomycin for 2 minutes. To induce mitochondrial Ca^2+^ uptake [Ca^2+^] in the superfusion medium was raised from 0 to 5 µM, still in the presence of the drugs. In *OPA1* silenced H295R cells Ca^2+^ uptake rate increased by a mean 30% (p = 0.034) as compared with the effect of control RNA and amounted nearly to the double of that measured in *Mfn1* silenced cells (p = 0.010, Figure 5). Applying the same protocol in HeLa cells, no difference in Ca^2+^ uptake rate was detected when [Ca^2+^]_c_ was raised to 2 µM but upon adding 5 µM Ca^2+^ a mean 55% increase in uptake rate was observed in *OPA1*-silenced cells as compared with the effect of *Mfn1* silencing (p =  0.00008). Moreover, OPA1-knockdown augmented Ca^2+^ uptake rate when compared to control RNA treatment (p = 0.00008, Figure 6). The 2-min depolarizing treatment did not change the immunoblot pattern of OPA1 (Figure 6).

### Pharmacological characterization of the Ca^2+^ transport mechanism in depolarized mitochondria

Mitochondrial Ca^2+^ uptake by MCU (in polarized mitochondria) and by the H^+^/Ca^2+^ antiporter (Letm1) is inhibited by Ruthenium Red. In permeabilized, *OPA1*-silenced H295R cells the drug (15 µM) strongly inhibited Ca^2+^ uptake rate (p = 0.00016). CGP-37157 (25 µM), an inhibitor of the mitochondrial Na^+^/Ca^2+^ exchanger exerted a similar effect (p = 0.00016). No inhibition was observed with cyclosporin A (10 µM), an inhibitor of the mitochondrial permeability transition pore (mPTP) (Figure S5 panel A and Table S1). Ruthenium Red reduced Ca^2+^ uptake rate to one fifth of the control (p = 0.00001) in permeabilized HeLa cells transfected with *OPA1* siRNA. CGP-37157 and cyclosporin A failed to exert any effect on Ca^2+^ uptake (Figure S5 Panel B and Table S1).

## Discussion

Mitochondrial Ca^2+^ accumulation occurs predominantly through the recently identified MCU protein [46], [47], an inwardly rectifying Ca^2+^ channel [44]. The uniporter does not function in depolarized mitochondria [60], under such conditions the mitochondrial Na^+^/Ca^2+^ exchanger (NCLX) and/or the Ca^2+^/H^+^ exchanger (Letm1) may be responsible for mitochondrial Ca^2+^ accumulation [49], [51], [61], [62]. An electronmicroscopic study detected the Na^+^/Ca^2+^ exchanger predominantly in the crista membrane [52] but no information is available for the localization of MCU or Letm1 within the IMM. The major purpose of the present study was to elucidate whether Ca^2+^ uptake occurs exclusively on the inner boundary membrane as suggested in the schemes of several recently published reviews (see Introduction) or it also takes place in the crista membrane [4], [6], [33]. Recalling that *OPA1*-knockdown increases the diffusibility of cytochrome *c* through the crista junctions [4], [6], [33] we presumed that if Ca^2+^ is sequestered through the crista membrane, in *OPA1*-silenced cells the transport should be enhanced.

In accordance with previous reports [19], [29], [30] silencing of *OPA1* induced fragmentation of mitochondria. It had to be considered that the fragmentation results in increased surface/volume ratio that in turn may lead to amplified increases in [Ca^2+^]_m_. Therefore cells transfected with *Mfn1* siRNA, also displaying mitochondrial fragmentation [26]–[28], rather than control RNA-treated cells were regarded as appropriate control.Significantly, shortening and circularity of mitochondria were comparable in the *OPA1* and *Mfn1* silenced groups.

In K^+^-stimulated H295R cells mitochondrial Ca^2+^ uptake was enhanced by *OPA1* knockdown as compared to control RNA or *Mfn1* siRNA-transfected cells. Stimulation of HeLa cells with histamine elicited cytosolic Ca^2+^ signal which was rapidly transferred into the mitochondrial matrix. Whereas *OPA1* siRNA had no obvious effect on the generation of mitochondrial Ca^2+^ signal in cells showing low cytosolic Ca^2+^ response, significantly bigger mitochondrial Ca^2+^ response and Ca^2+^ uptake rate were attained after *OPA1* knockdown in cells showing high cytosolic response. We presume that at higher Ca^2+^ load the access of Ca^2+^ to those transporters that are localized in the crista membrane becomes the rate-limiting factor of the transport. Confirming previous reports [11], [24], [63], knockdown of *OPA1* but not that of *Mfn1* resulted in mitochondrial depolarization implying that in *OPA1* silenced cells Ca^2+^ uptake rate increased in spite of smaller driving force. The above data indicate that OPA1 attenuates mitochondrial Ca^2+^ signaling in intact cells.

In order to ensure stable and identical driving force in each group Ψ_m_ was dissipated in permeabilized cells. Permeabilization of control RNA-transfected cells resulted in the formation of toroids, probably due to a partial dissociation of the mitochondria from the microtubules [64]. The permeabilization did not cause any detectable morphological change of the fragmented mitochondria in siRNA-transfected cells (not shown). After complete depolarization [Ca^2+^] was raised to 2 or 5 µM, ensuring that the concentration gradient of Ca^2+^ should be the only driving force of the transport [65]. ATP depletion [66] as well as protonophores may evoke proteolysis of OPA1 [21], [22], [67], however, this did not occur within 2 minutes in the depolarizing medium. Elevation of [Ca^2+^] to 5 µM induced an immediate mitochondrial Ca^2+^ influx in permeabilized H295R cells and Ca^2+^ uptake rate was significantly increased after silencing *OPA1*. Silencing also augmented mitochondrial Ca^2+^ accumulation in permeabilized HeLa cells exposed to 5 rather than to 2 µM Ca^2+^. This phenomenon resembles the observation in intact cell where the signal transfer was accelerated by *OPA1* siRNA only in cells displaying high cytosolic Ca^2+^ signal.

Ca^2+^ uptake may have been enhanced in *OPA1*-silenced cells due to hyperpolarization, increased expression or higher Ca^2+^ affinity of the transporter or increased access of Ca^2+^ to the transporter. *OPA1* siRNA reduces Ψ_m_ and therefore the driving force of Ca^2+^ uptake. (As to the effect of mitochondrial pH, it should be recalled that the protonophore FCCP was present in all the experiments on permeabilized cells.) Had the density or affinity of the transporter increased, enhanced Ca^2+^ accumulation could be expected even in the presence of lower [Ca^2+^]_c_ but this was not the case in HeLa cells. Therefore it can be considered that, when [Ca^2+^]_c_ is high, Ca^2+^ supply of the transporters located in the crista membrane is a limiting factor. Enhanced access of Ca^2+^ to these transporters in *OPA1*-silenced cells then augments the efficiency of the Ca^2+^ uptake process. Nevertheless, the possibility should be kept in mind that if ablation of *OPA1* evokes the relocation of the transporter molecules from the crista into the boundary membrane, mitochondrial Ca^2+^ metabolism would alter similarly to the present observations. Unfortunately, this is difficult to prove or disprove at this stage.

Which transport mechanism is located in the crista membrane? The predominant mechanism of Ca^2+^ uptake in polarized mitochondria is the MCU [41]–[43]. The Ca^2+^ transporting capacity of the supposedly electrogenic H^+^/Ca^2+^ antiporter [51] as compared to that of MCU, is negligible [68]. The kinetics of Ca^2+^ uptake in our cells argues against any role of the *rapid mode* of Ca^2+^ uptake [69]. The enhancement of mitochondrial Ca^2+^ uptake in intact, *OPA1*-silenced cells is compatible with the presence of MCU in the crista membrane.

The transport mechanism was further analyzed in *OPA1* siRNA-transfected, permeabilized cells after depolarizing the mitochondria. MCU is inactive in depolarized mitochondria [60]. Ca^2+^ uptake by depolarized mitochondria in permeabilized HeLa cells was reduced by Ruthenium Red. The Ca^2+^/H^+^ exchanger Letm1 is expressed in HeLa cells and is inhibited by Ruthenium Red [51]. Ca^2+^ uptake was not influenced by CGP-37157, an inhibitor of the Na^+^/Ca^2+^ antiporter. In the excitable cell type H295R Ruthenium Red almost completely abolished Ca^2+^ uptake and CGP-37157 also exerted a strong inhibition [70]. The pharmacological data suggest that, in addition to MCU, both the Ca^2+^/H^+^ and Na^+^/Ca^2+^ exchanger in H295R cells and the former one in HeLa cells participate in the enhanced Ca^2+^ uptake after knockdown of *OPA1*. In this respect it is worthwhile to recall that both antiporters are present in the adrenal cortex [70]. Cyclosporin A, an inhibitor of mPTP had no effect in either cell type. Indeed, in lack of contact between the crista and outer mitochondrial membranes mPTP may not be directly involved in Ca^2+^ uptake through the crista membrane.In a study on murine retinal ganglion cells, transiently stimulated with K^+^, the subsequently added protonophore induced bigger cytosolic Ca^2+^ signals in *OPA1*-silenced than in control cells [11]. The data which show the resultant of mitochondrial Ca^2+^ release and elimination of cytosolic Ca^2+^ by Ca.ATPases, can be attributed to changes in mitochondrial Ca^2+^ accumulation. However, in lack of appropriate control with fragmented mitochondria, the role of changes in various transports processes (e.g. rate of mitochondrial depolarization, rate of Ca^2+^ pumping) should also be considered. Our direct measurements of mitochondrial Ca^2+^ metabolism not only demonstrated the increased *rate* of Ca^2+^ uptake in *OPA1*-silenced cells but also excluded the role of mitochondrial membrane potential in this action of the protein and suggested the presence of Ruthenium Red-sensitive transport mechanisms in the crista membrane.

Summarizing, the reduction of OPA1 expression results in enhanced mitochondrial Ca^2+^ uptake rate and augments the mitochondrial Ca^2+^ signal. These results unambiguously demonstrate that OPA1 restrains mitochondrial Ca^2+^ uptake. On the basis of published data we attribute the uptake attenuating effect of OPA1 to decreased permeation of Ca^2+^ through the junction of the cristae. It follows that enhanced Ca^2+^ uptake in the absence of normal OPA1 function may be an aggravating component of OPA1-related diseases. Therefore OPA1 may be a target of factors modifying mitochondrial Ca^2+^ handling.

## Materials and Methods

### Cell culture and transfection

H295R cells (CRL-2128, ATCC, Manassas, VA) were grown in DMEM/Ham's F12 (1∶1 v/v) containing 1% ITS^+^, 2% UltroSer G, 100 U/ml penicillin and 100 µg/ml streptomycin. HeLa cells (CLL-2, ATCC, Manassas, VA) were grown in DMEM containing 10% heat-inactivated FBS, 100 U/ml penicillin and 100 ug/ml streptomycin. Passage numbers 3–20 were used.

Cells (about 4*10^4^ H295R or 10^4^ HeLa) were plated onto 24-mm diameter circular glass coverslips on day 1. For plasmid transfection we used 1 µg DNA (or 2 µg in case of H295R transfection with 4mt-D2-cpV) with transfection reagent (2 µl Lipofectamine 2000 for H295R or 2–3 µl FuGENE-HD for HeLa cells) in 1.1 ml OPTI-MEM medium. The transfection was performed on day 2 or 3. For silencing *OPA1* a mixture of three siRNA species (1299003) was applied, for that of *Mfn1* the product 5141600 and for control a non-silencing RNA with appropriate GC content (12935400 or 129305200), all from Invitrogen (Paisley, UK). Transfection took place on day 2, 40 pmol siRNA were added with 1 µl Lipofectamine RNAiMAX in 1.1 ml OPTI-MEM medium. When RNA was co-transfected with the plasmid coding for the Ca^2+^ sensitive protein, the protocol for plasmid transfection was applied. In experiments on intact H295R cells RNA transfection was repeated on day 3. The transfection reagents were purchased from Invitrogen, with the exception of FuGENE-HD (Roche, Mannheim, Germany). The experiments were conducted 56–76 hours after the first transfection.

### Immunoblotting

10^5^ cells cultured in 24-mm diameter dishes were suspended in ice-cold lysis buffer (100 mM NaCl, 30 mM HEPES pH 7.4, 0.2% Triton X-100, 20 mM NaF, 2.5 mM Na-EGTA, 2.5 mM Na-EDTA, 10 mM benzamidine, 0.075 U/ml Aprotinin, 1∶100 Sigma Mammalian Protease Inhibitor Cocktail, 1 mM sodium-vanadate, 10 mM PMSF). Insoluble fraction was removed with centrifugation. Protein concentration was measured with Bradford or BCA assay. The supernatant was completed with 1/3 volume reducing buffer (125 mM TrisCl pH 6.8, 40% glycerol, 20% mercaptoethanol, 0,02% bromophenolblue, 280 mM SDS). Samples were run on 8% SDS-PAGE and transferred onto nitrocellulose membrane (pore size: 0.45 µm). The membrane was incubated with 3% milk powder+0.1% Tween 20 in PBS to block nonspecific binding sites. Detection of OPA1 was performed with anti-OPA1 mouse monoclonal antibody (Cat. No 612606, BD Bioscience, Franklin Lakes, NJ) followed by incubation with anti-mouse immunoglobulin-horseradish-peroxidase conjugate (1∶2000) (GE Healthcare, Amersham, UK). Protein disulphide isomerase was used as loading control (anti-PDI antibody: ab2792, Abcam, Cambridge, UK).

### Confocal microscopy

The rate of mitochondrial Ca^2+^ uptake and mitochondrial membrane potential were examined with confocal microscopy. Cells plated onto glass coverslips were placed on the stage of Zeiss LSM510 confocal laser scanning microscope equipped with a 40×/1.3 oil immersion objective (Plan-Neofluar Zeiss). Mt-inv-Pericam (gifted by Prof. A. Miyawaki, Saitama, Japan) was excited at 488 nm; emitted light was filtered using BP 500–550 nm emission filter. Rhod-2 (3 µM for 15 min) was excited at 543 nm; the emitted light was filtered using LP 560 filter. The optical slice was 5 µm, image acquisition frequency in the Ca^2+^ uptake experiments was 1-0.2 Hz. The experiments were performed at room temperature. The solutions were applied with a solenoid valve-equipped, gravity-driven superfusion system, terminating at ∼2 mm from the selected cells. Flow rate was ∼1 ml/min. Fluorescence intensity was normalized to the intensity measured before stimulation. The initial linear section of the normalized curves was regarded as rate of Ca^2+^ uptake. Uptake rate was expressed as (ΔF/F_0_)/Δt (Rhod-2) or ((ΔF−F_min_)/(F_0_−F_min_))/Δt (mt-inv-Pericam) where F/F_0_ is fluorescence intensity (F) related to that measured during the control period (F_0_) and F_min_ is the fluorescence measured at saturating [Ca^2+^]_c_.

Changes in Ψ_m_ were followed with TMRE or TMRM. Cells preloaded with the dye were incubated in the presence of 25 nM dye. TMRE or TMRM was excited at 543 nm, emitted light was filtered using LP 560 emission filter. Alternatively, Ψ_m_ was examined with JC-1 (30-min loading with 1 µM at 37°C followed by 10-min incubation in the modified Krebs-Ringer solution without dye). JC-1 was monitored in “Multi Track” mode, excitation wavelengths of 488 nm and 543 nm were applied, emitted light was separated with a beam splitter cutting at 545 nm and emission filters BP 500–550 nm (green) and LP560 nm (red), resp., were used. The TMRE or TMRM fluorescence intensities and the ratios of red to green intensities in JC-1 experiments were normalized with that measured after depolarizing the mitochondria with FCCP.

For the morphological analysis of mitochondria the cells were transfected with GFP fused with human cytochrome *c* oxidase VIII target sequence. The length and circularity ((4π(area)/(perimeter)^2^) of mitochondria were analyzed with ImageJ 1.6.0, as suggested [71]. (The major steps were as follows: background substraction – deconvolution – smoothing – substraction of a *Mexican hat* convolved duplicate – threshold – scale setting – analysis of particles.)

### FRET measurements with fluorescent wide-field microscopy

Mitochondrial [Ca^2+^] was monitored with the appropriately targeted Ca^2+^ sensitive fluorescent proteins 4mt-D1-cpV (a gift from Prof. T. Pozzan, Padova, Italy) or 4mt-D2-cpV. In order to prepare 4mt-D2-cpV we cut out the 4-mt domain from 4mt-D1-cpV cloned in pcDNA3.1 with HindIII and inserted it in site of the 2-mt domain of 2mt-D2-cpV (a gift from Dr. A.E. Palmer, Boulder, CO) also cloned in pcDNA3.1.

Fluorescence intensity measurements were performed on an inverted microscope (Axio Observer D1, Zeiss) equipped with a 40×1.4 oil immersion objective (Fluar, Zeiss) and a Cascade II camera (Photometrics). Excitation wavelengths were set by a random access monochromator connected to a xenon arc lamp (DeltaRAM, Photon Technology International). For ratiometric FRET measurements of mitochondrially targeted D1 or D2-cpV excitation wavelength of 430 nm was selected along with a Dual-View emission splitting system (505dcxr, 480/30 and 535/30; Photometrics) enabling the acquisition of simultaneous donor and raw FRET emissions. Alternately cytosolic [Ca^2+^] was estimated with Fura-2 or Fura-FF, excited with 340 and 380 nm and using the above emission splitting system. Images were acquired every 5 seconds with the MetaFluor software (Molecular Devices) and MetaMorph was used for data analysis. FRET and Fura ratios were calculated after background subtraction; ratio of raw FRET acceptor (Venus) and donor (Cerulean) fluorescence or that of 340 nm/380 nm were used, respectively. Ratios were normalized to the control period (R_0_). Mitochondrial Ca^2+^ uptake rate was expressed as (ΔR/R_0_)/Δt. Measurements were performed at 31°C.

### Solutions

Incubation of intact cells was performed in a modified Krebs-Ringer solution containing 3.6 mM K^+^, 1.2 mM Ca^2+^, 0.5 mM Mg^2+^, 10 mM Hepes and 2 mM HCO_3_^−^ (pH 7.4). In experiments studying the effect of K^+^, NaCl was partly replaced with N-methyl-d-glucamine or K^+^ in order to maintain Na^+^ and osmotic concentrations constant. Permeabilization was carried out in a cytosol-like medium (117 mM KCl, 6 mM NaCl, 1 mM KH_2_PO_4_, 2 mM Na^+^ pyruvate, 2 mM Na^+^ succinate (for HeLa) or 2 mM Na^+^ isocitrate (for H295R), 2 mM K^+^ADP, 2 mM EGTA, 10 mM K^+^HEPES or 10 mM K^+^MOPS) with 25 µg/ml digitonin at room temperature for 10 minutes. To adjust the [Ca^2+^] and [Mg^2+^] of the cytosol-like media, EGTA, HEDTA, CaCl_2_, MgCl_2_ and ADP were used as calculated by the Chelator software [72]. The [Ca^2+^] of the applied solutions was checked with a Ca^2+^ selective electrode (Orion, Cambridge, UK). Calculated [Mg^2+^] was 0.5 mM.

### Statistics

Means + S.E.M. are shown. All the experiments were performed on at least two different cell passages. For estimating significance of differences, Student's unpaired *t*-test (with or without Welch correction), one-way ANOVA, Tukey's or unequal N HSD post hoc tests or Kruskal-Wallis test were used, as appropriate. Data were analyzed with Statistica 9.

## Supporting Information

Figure S1 **Morphology of the mitochondria of RNA-transfected H295R cells.** The cells were transfected with control RNA (**A**), *Mfn1* siRNA (**B**) or *OPA1* siRNA (**C**) on the day following plating (day 2) and with mitochondrially targeted GFP on day 3. Confocal microscopy was performed on day 4. The framed areas are shown in the right-hand column; zoom: 4×. Optical slice thickness was 1 µm. Bars, 10 µm.
(TIF)

Figure S2 **Morphology of the mitochondria of RNA-transfected HeLa cells.** The cells were transfected with control RNA (**A**), *Mfn1* siRNA (**B**) or *OPA1* siRNA (**C**) on the day following plating (day 2) and with mitochondrially targeted GFP on day 3. Confocal microscopy was performed on day 5. The framed areas are shown in the right-hand column; zoom: 4×. Optical slice thickness was 1 µm. Bars, 10 µm.
(TIF)

Figure S3 **Morphometry of the mitochondria of RNA-transfected cells.** For the experimental protocol see the legend of Figure S1 (H295R) or 2 (HeLa). The histograms show the length and circularity of mitochondria in H295R cells (**A** and **B**, resp.) and in HeLa cells (**C** and **D**, resp.). Analysis was performed with ImageJ 1.6.0, as suggested [71].
(TIF)

Figure S4 **Mitochondrial membrane potential of RNA-transfected HeLa cells.** Transfection with control RNA, *Mfn1* or *OPA1* siRNA was performed on the day following plating. Three days later Ψ_m_ was estimated applying TMRM in H295R cells and TMRE or JC-1 in HeLa cells, respectively. TMRM and TMRE fluorescence or JC-1 ratio (red/green) over the mitochondrial region were normalized to that measured after depolarisation with FCCP. Means + SEM are shown, the number of observations is shown within the columns.
(TIF)

Figure S5 **Pharmacological characterization of the Ca^2+^ transport mechanism in depolarized mitochondria.** H295R cells (**A**) or HeLa cells (**B**) were transfected with *OPA1* siRNA 1 day after plating (day 2). On day 5 the cells were loaded with Rhod-2 AM, permeabilized and superfused with a cytosol-like medium. Ψ_m_ was dissipated (see legend of Figure 5) and then [Ca^2+^] was raised from 0 to 5 µM. Rhod-2 fluorescence data were evaluated as described in *Methods*. Fifteen µM Ruthenium Red (RR), 25 µM CGP-37157 (CG) and 10 µM cyclosporine A (Cy), added alone or in combination, were present from the beginning of permeabilization. The means + SEM of the slopes of the initial increase in normalized Rhod-2 fluorescence are shown. The number of observations is shown within the columns. For the significance of differences see Table S1.
(TIF)

Table S1 **Significance of differences for the experiments shown in Figure S5.** C: control, RR: Ruthenium Red, CG: CGP-37157, Cy: cyclosporine A.
(DOC)
