# Supplementary figures and images for: The Effect of OPA1 on Mitochondrial Ca2+ Signaling
Source: PLoS One. 2011 Sep 29;6(9):e25199. doi: 10.1371/journal.pone.0025199 (PMC3182975; doi:10.1371/journal.pone.0025199)

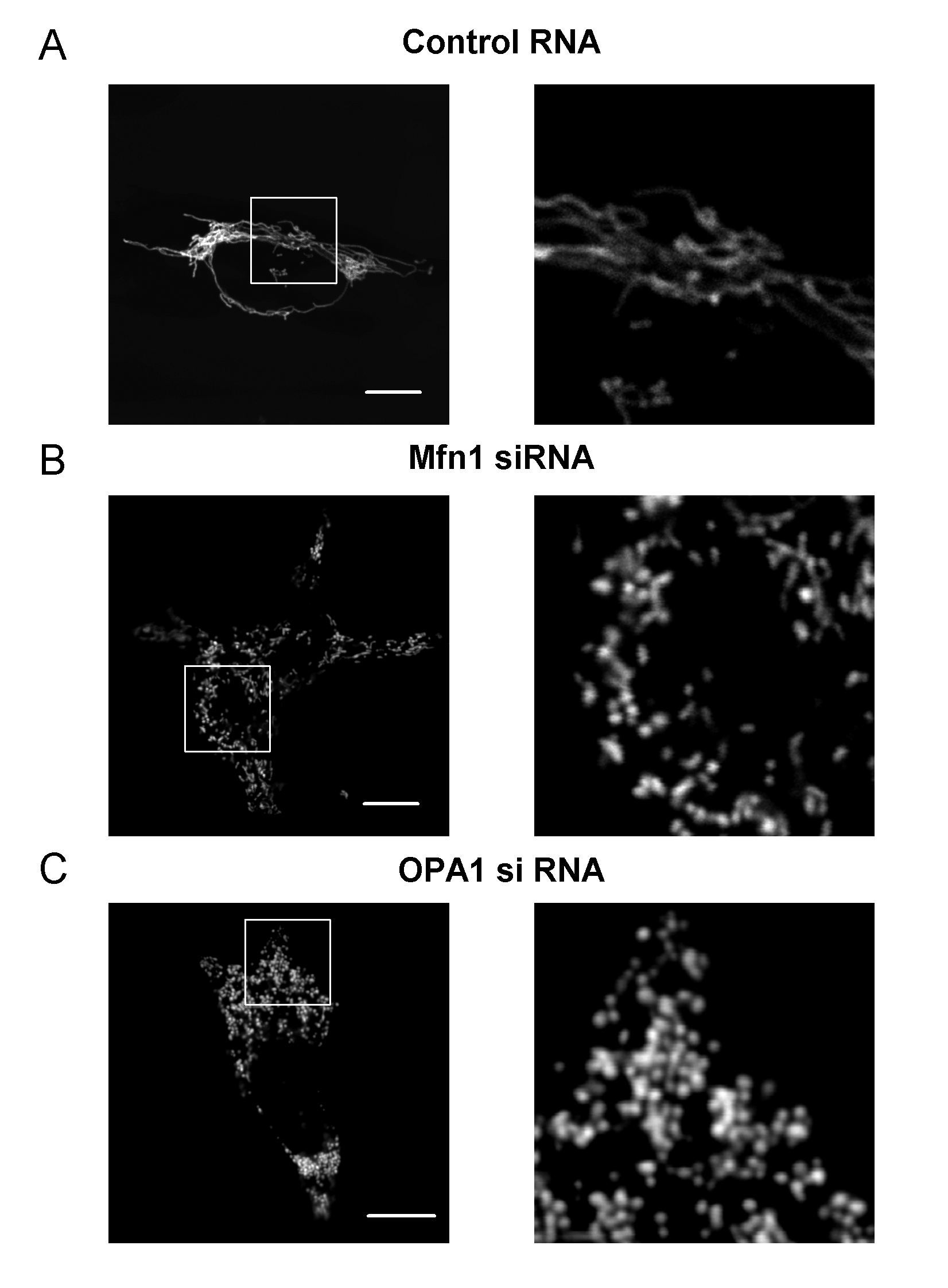

Supplement: Figure S1 — Morphology of the mitochondria of RNA-transfected H295R cells. The cells were transfected with control RNA (A), Mfn1 siRNA (B) or OPA1 siRNA (C) on the day following plating (day 2) and with mitochondrially targeted GFP on day 3. Confocal microscopy was performed on day 4. The framed areas are shown in the right-hand column; zoom: 4×. Optical slice thickness was 1 µm. Bars, 10 µm. (TIF) [file pone.0025199.s001.tif]

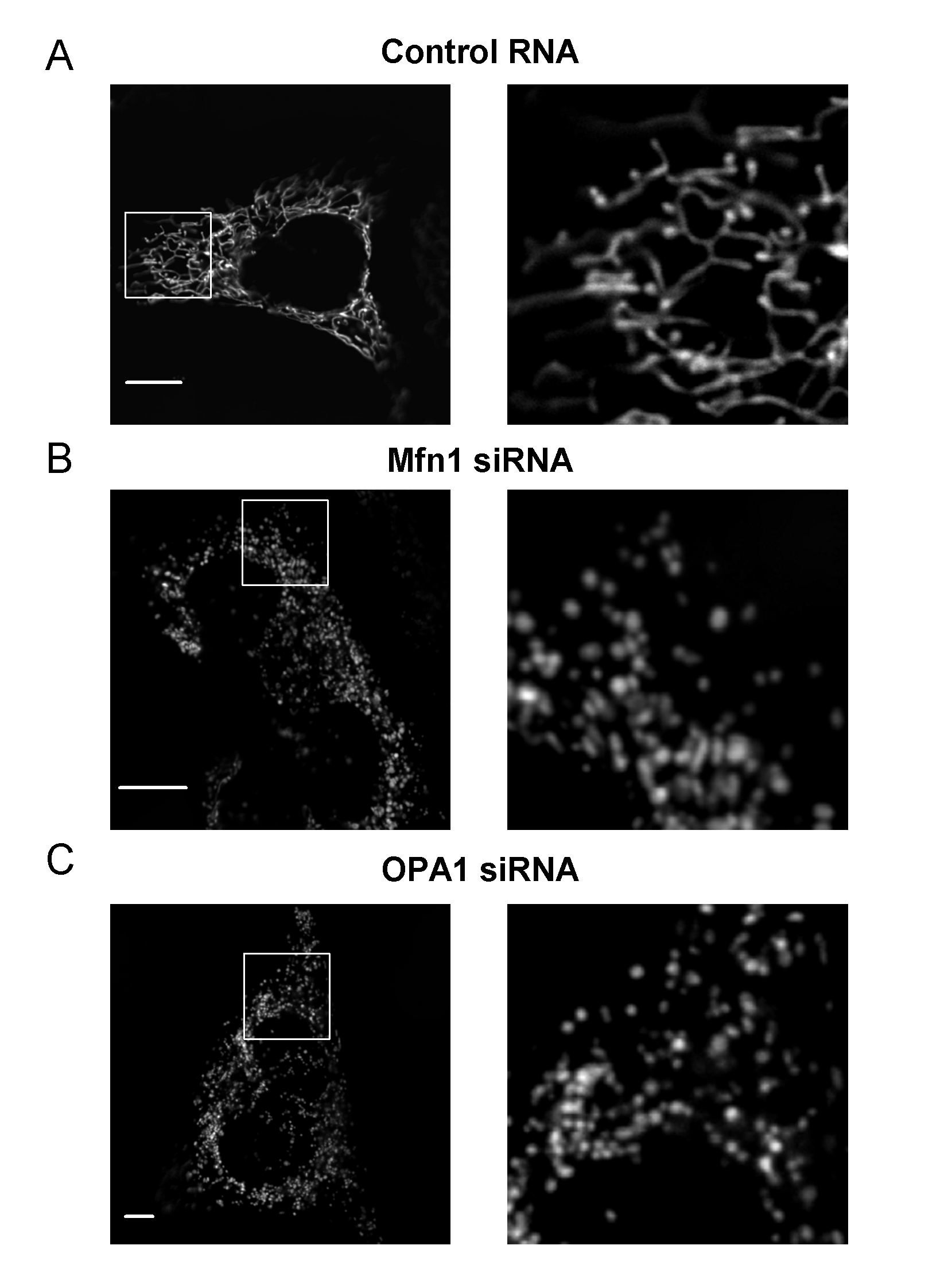

Supplement: Figure S2 — Morphology of the mitochondria of RNA-transfected HeLa cells. The cells were transfected with control RNA (A), Mfn1 siRNA (B) or OPA1 siRNA (C) on the day following plating (day 2) and with mitochondrially targeted GFP on day 3. Confocal microscopy was performed on day 5. The framed areas are shown in the right-hand column; zoom: 4×. Optical slice thickness was 1 µm. Bars, 10 µm. (TIF) [file pone.0025199.s002.tif]

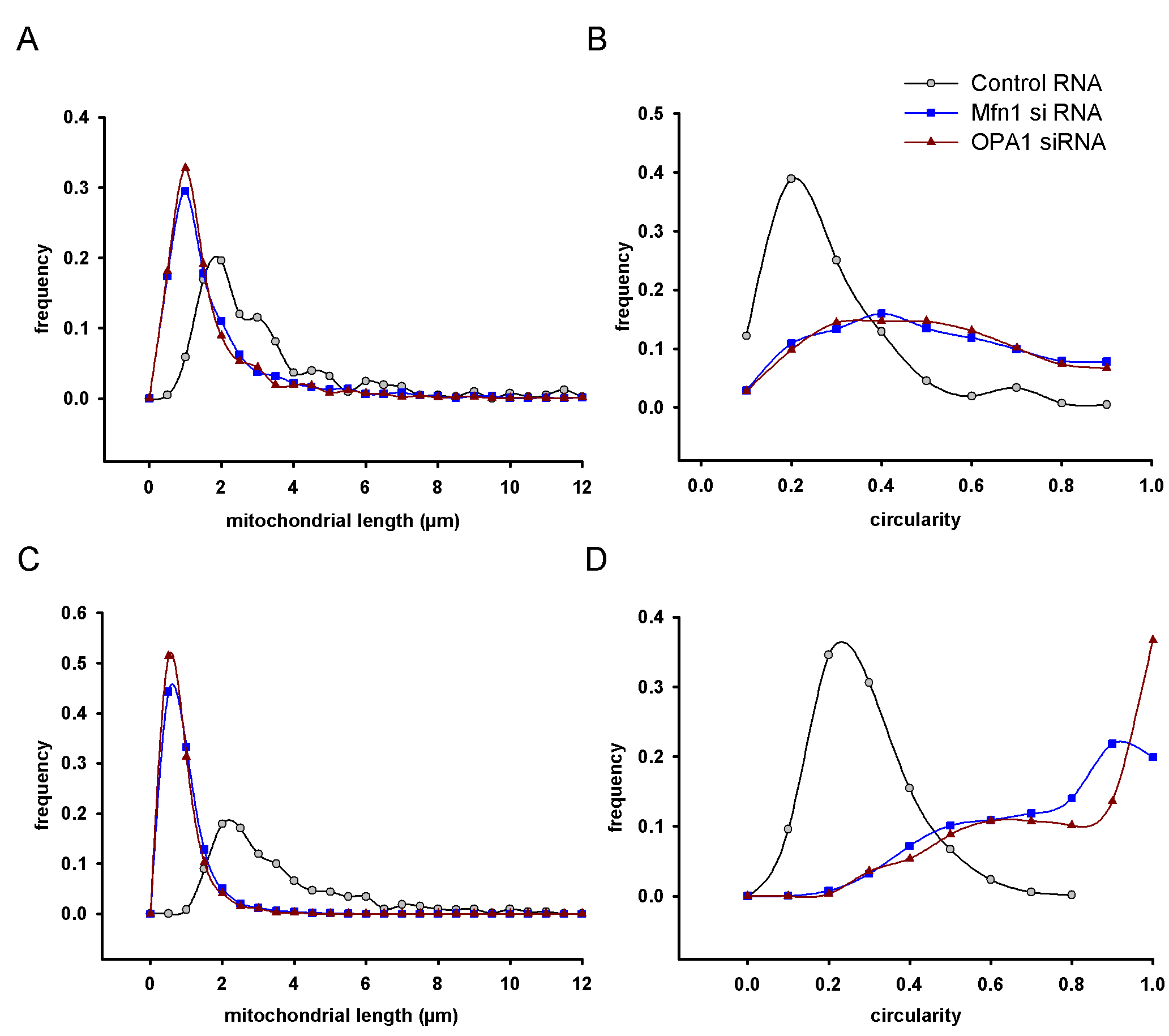

Supplement: Figure S3 — Morphometry of the mitochondria of RNA-transfected cells. For the experimental protocol see the legend of Figure S1 (H295R) or 2 (HeLa). The histograms show the length and circularity of mitochondria in H295R cells (A and B, resp.) and in HeLa cells (C and D, resp.). Analysis was performed with ImageJ 1.6.0, as suggested [71]. (TIF) [file pone.0025199.s003.tif]

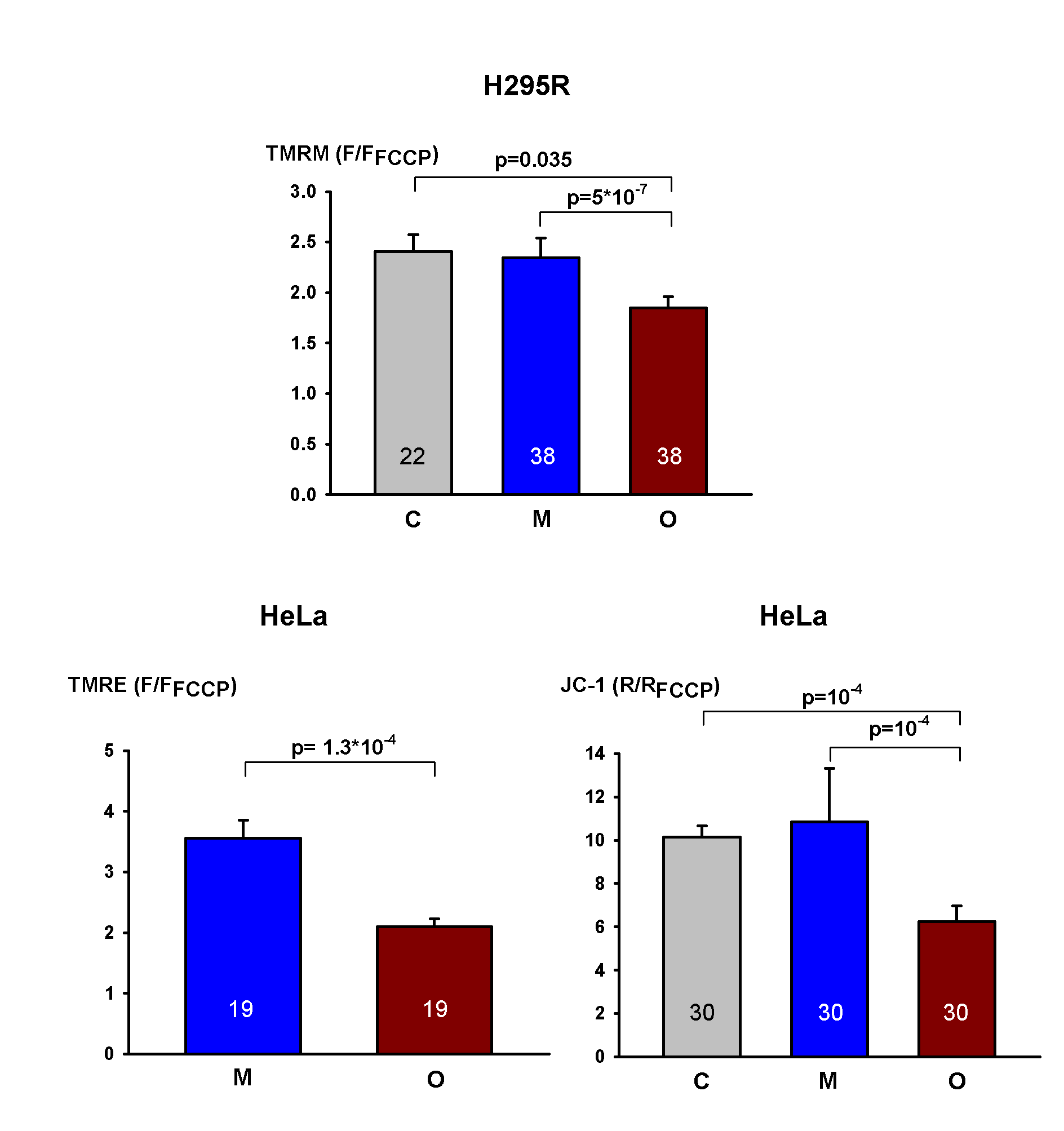

Supplement: Figure S4 — Mitochondrial membrane potential of RNA-transfected HeLa cells. Transfection with control RNA, Mfn1 or OPA1 siRNA was performed on the day following plating. Three days later Ψm was estimated applying TMRM in H295R cells and TMRE or JC-1 in HeLa cells, respectively. TMRM and TMRE fluorescence or JC-1 ratio (red/green) over the mitochondrial region were normalized to that measured after depolarisation with FCCP. Means + SEM are shown, the number of observations is shown within the columns. (TIF) [file pone.0025199.s004.tif]

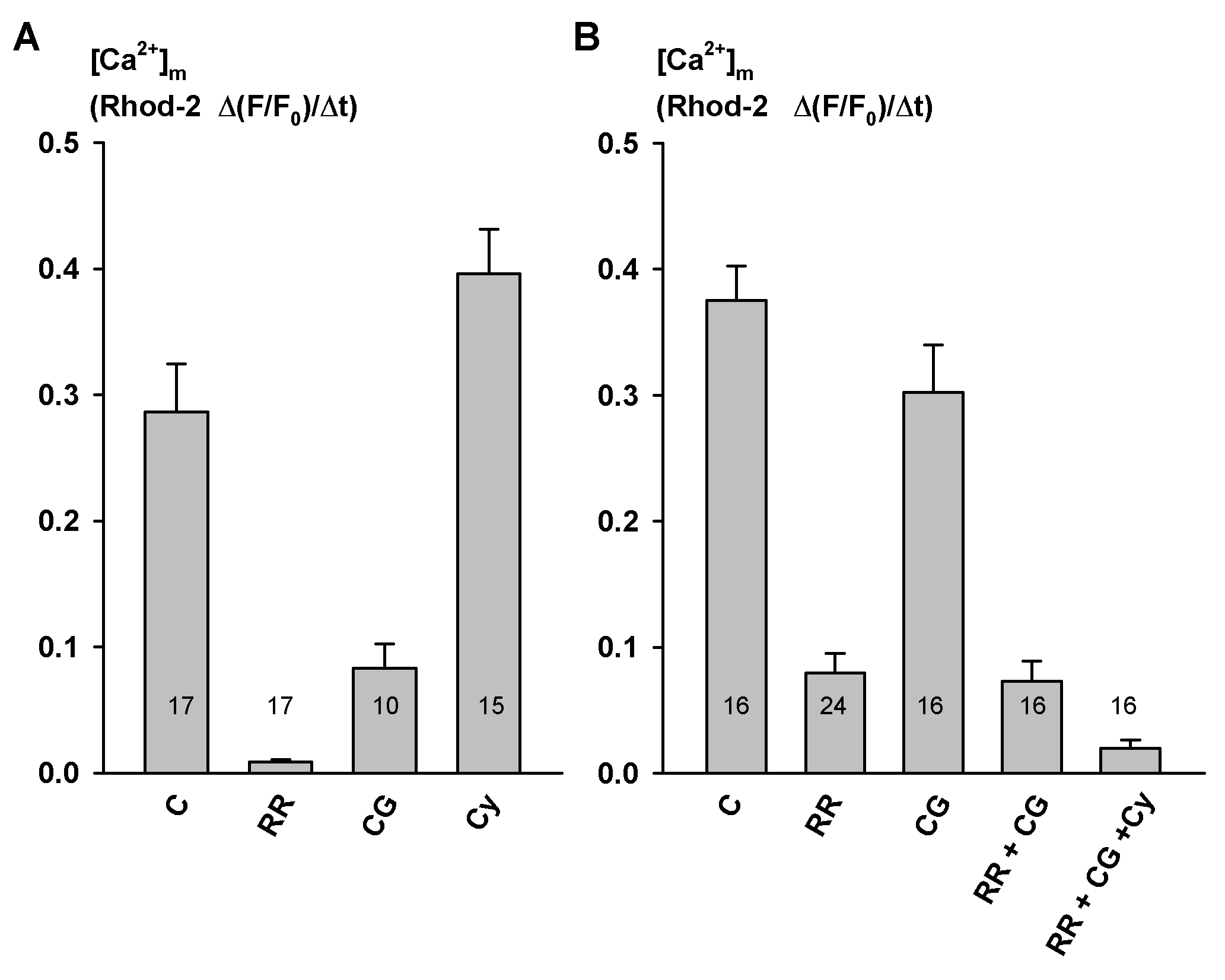

Supplement: Figure S5 — Pharmacological characterization of the Ca2+ transport mechanism in depolarized mitochondria. H295R cells (A) or HeLa cells (B) were transfected with OPA1 siRNA 1 day after plating (day 2). On day 5 the cells were loaded with Rhod-2 AM, permeabilized and superfused with a cytosol-like medium. Ψm was dissipated (see legend of Figure 5) and then [Ca2+] was raised from 0 to 5 µM. Rhod-2 fluorescence data were evaluated as described in Methods. Fifteen µM Ruthenium Red (RR), 25 µM CGP-37157 (CG) and 10 µM cyclosporine A (Cy), added alone or in combination, were present from the beginning of permeabilization. The means + SEM of the slopes of the initial increase in normalized Rhod-2 fluorescence are shown. The number of observations is shown within the columns. For the significance of differences see Table S1. (TIF) [file pone.0025199.s005.tif]
