# Supplementary material for: The Effect of OPA1 on Mitochondrial Ca2+ Signaling
Source: PLoS One. 2011 Sep 29;6(9):e25199. doi: 10.1371/journal.pone.0025199 (PMC3182975; doi:10.1371/journal.pone.0025199)
Supplement: Table S1 — Significance of differences for the experiments shown in Figure S5. C: control, RR: Ruthenium Red, CG: CGP-37157, Cy: cyclosporine A. (DOC) [file pone.0025199.s006.doc]

| **H295R** | C | RR | CG | Cy |
| --- | --- | --- | --- | --- |
| C |  | 1.6*10^-4^ | 1.6*10^-4^ |  |
| RR | 1.6*10^-4^ |  | 1.6*10^-4^ | 1.6*10^-4^ |
| CG | 1.6*10^-4^ | 1.6*10^-4^ |  | 1.6*10^-4^ |
| Cy |  | 1.6*10^-4^ | 1.6*10^-4^ |  |

| **HeLa** | C | RR | CG | RR + CG | RR+CP+Cy |
| --- | --- | --- | --- | --- | --- |
| C |  | 1.3*10^-5^ |  | 1.2*10^-4^ | 2.1*10^-9^ |
| RR | 1.3*10^-5^ |  | 7.4*10^-4^ |  |  |
| CG |  | 7.4*10^-4^ |  | 3.4*10^-3^ | 2.8*10^-7^ |
| RR + CG | 1.2*10^-4^ |  | 3.4*10^-3^ |  |  |
| RR+CG+Cy | 2.1*10^-9^ |  | 2.8*10^-7^ |  |  |
